# Green Synthesis of Copper Nanoparticles utilising the Maillard Reaction

**DOI:** 10.1002/chem.202404314

**Published:** 2025-02-12

**Authors:** Lukas Mielewczyk, Virginia Liebscher, Julia Grothe, Stefan Kaskel

**Affiliations:** ^1^ Inorganic Chemistry I Technische Universität Dresden Bergstraße 66 01069 Dresden

**Keywords:** Amadori rearrangement, arginine, chemical reduction, copper, nanoparticles

## Abstract

A new approach for the fabrication copper nanoparticles by a wet chemical reduction method is reported. The natural resources arginine as amino compound and several monosaccharides (xylose, ribose, galactose and glucose) react characteristically performing an Amadori rearrangement followed by a Maillard type reaction. This reaction carried out in an aqueous solution ensures an environmentally friendly way of reducing copper(II) ions leading to the formation of the desired nanoparticles. By changing the concentration of the amino acid, simultaneously acting as a complexing agent, it is possible to tune the size of the resulting nano particles to a certain degree down to 3 nm. This nontoxic and facile preparation route with quantitative yield opens a wide field of applications ranging from electronics to medical approaches.

## Graphical abstract

This paper presents a green synthesis method for copper nanoparticles using the Amadori rearrangement followed by the Maillard reaction. Naturally derived reagents like monosaccharides (xylose and galactose) and arginine reduce copper ions in an aqueous solution. The nanoparticle size, adjustable by arginine concentration, ranges from in the lowest regime between 3–16 nm.

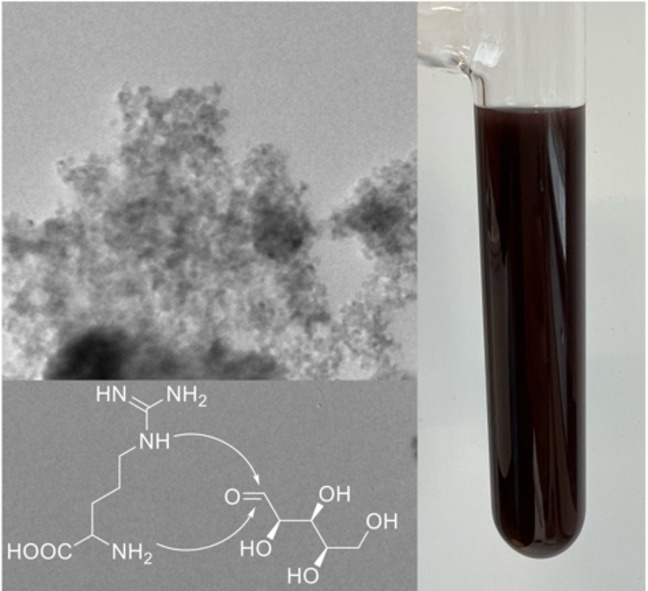

## Introduction

The preparation of copper nanoparticles has gained much attention for various applications in recent years. Because of the higher demand, research concerning the use of greener synthesis methods is a key target for the future. Copper nanoparticles, like all nanoparticles in general, show unique properties in comparison to the bulk material due to their small size, including optical properties[^1^, ^2^, ^3^] and high thermal conductivity.^[4]^ Depending on the resulting properties several fields of applications can be considered. For example, copper nanoparticles are widely used in printed electronic technologies[^5^, ^6^, ^7^, ^8^] where these particles are processed in nanoinks.[^9^, ^10^] Typical methods to produce nanoparticles can be divided into physical and chemical approaches. Physical methods include for example pulse laser ablation,^[11]^ vacuum vapour deposition,^[12]^ pulse wire discharge,^[13]^ mechanical milling.^[14]^ However, due to the complex devices and conditions required for these techniques, they usually go along with high energy consumptions. Further, chemical methods like polyol reduction,^[15]^ microemulsion techniques,^[16]^ sonochemical reduction,^[17]^ electrochemical methods,^[18]^ hydrothermal^[19]^ and chemical reduction^[20]^ are used. Chemical reduction in particular is probably one of the easiest methods, because usually just basic laboratory equipment is used during the reaction.^[21]^ Traditionally, the preparation of nanoparticles via chemical reduction has been performed with hazardous reducing agents such as hydrazine,^[22]^ borohydrides^[23]^ or formaldehyde.^[24]^ Also, costly and often toxic protection agents are necessary.[^25^, ^26^, ^27^, ^28^, ^29^, ^30^] Until today, these harsh conditions are required, since most milder reducing agents result in pure copper(I) oxide or mixed phases with a high content of copper(I) oxide.[^31^, ^32^] Additionally, the use of organic solvents and surfactants goes along with a more difficult waste issue. Therefore, milder reaction conditions^[33]^ or the use of natural or naturally derived products were focused in order to achieve methods with non‐toxic educts and products during the process. One approach is the use of plant‐derived substances (biomass). These types of syntheses have been summed up as biogenic reduction methods.[^34^, ^35^] Other methods which are not directly dependent on mostly locally limited plants fall back on reagents which can be naturally sourced or could be released into the environment without harm. Many published reactions focus on the exchange of a specific step in the synthesis,^[36]^ hence remaining steps still show a certain toxicity. Therefore, some research was conducted to find more sustainable methods for the preparation.^[37]^ in this work we aimed to present an improved reaction for the reduction of copper. Here, environmentally friendly educts like monosaccharides and amino acids are used to perform the reaction. In an aqueous reaction mixture, the added copper salt is reduced only by the addition of a sugar as a reducing agent.

## Results and Discussion

Basis of this work is a previous publication in which the formation of copper coatings on ceramic particles with the use of sugars and sodium hydroxide has been investigated. Specifically, the influence of different amino acids in the Maillard reaction has been addressed in this work.^[38]^ The aim of the work presented here is to achieve the preparation of pure copper particles in the nanometer scale.

**Figure 1:**
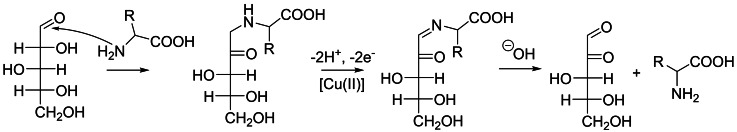
Proposed mechanism of the Amadori rearrangement where the sugar reacts with an amino acid. The resulting Amadori compound can reduce copper(II) ions. The amino acid can be restored by a condensation reaction induced by a base.

The size of nanoparticles is controlled via supersaturation leading to a high amount of crystal nuclei. The more crystal nuclei are present in a solution with a certain concentration the smaller are the resulting particles. This correlation follows the published work of LaMer^[39]^ concerning the nucleation and growth of nanoparticles. Following these concepts, it was crucial to improve the formation of the nuclei in solution, while in the previous work nucleation was initiated by complexation on a functionalised surface. In order to not deteriorate the atomic efficiency significantly, no other component than the previously introduced ones should be added, namely a sugar as reducing (structure in **Fig S1**) agent, an amino acid as amino compound, sodium hydroxide and the copper source (Figure 1). Since there are only these four compounds there is not much parameter space for tailoring the reaction conditions. The amino acid used for the reaction has a significant impact on the results. Many of the amino acids with the capability of complexing copper ions already have been tested..^[38]^ Another promising compound which was not tested yet is arginine, containing a chain which is functionalised with a guanidinium group. This not only should be able to complex copper ions during the reaction,^[40]^ but also influence the amount of crystal nuclei created before the particle growth just by varying the amount. For this, several concentrations of arginine were tested to see if a size change is observable.

**Figure 2:**
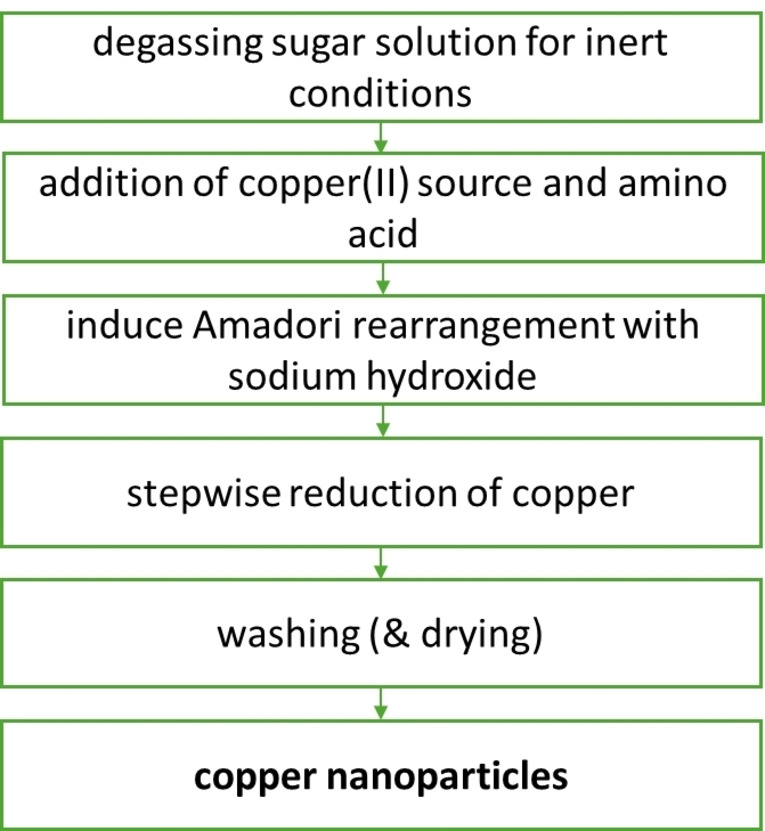
Proposed mechanism of the Amadori rearrangement where the sugar reacts with an amino acid. The resulting Amadori compound can reduce copper(II) ions. The amino acid can be restored by a condensation reaction induced by a base.

**Figure 3:**
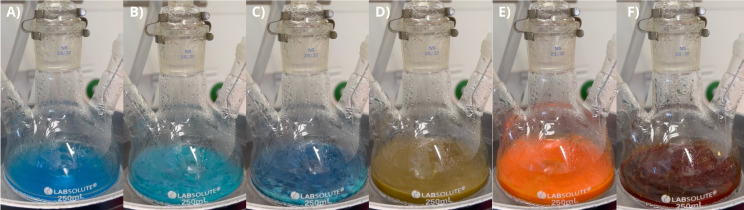
Images of the visible colour change after starting the reaction with sodium hydroxide addition. Starting with A) the copper(II) solution with arginine, B) the formation of light blue copper(II) hydroxide precipitate, C) the formation of the tetrahydroxy copper(II) complex, D) and E) formation of eventually copper(I) oxide and F) the final brown reaction mixture due to Maillard products.

**Figure 4:**
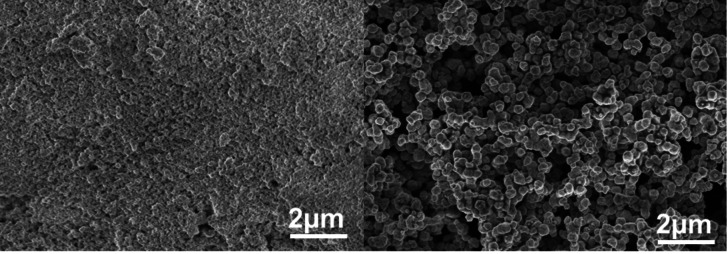
SEM images of the copper powder resulting from the reaction with the highest arginine content 27 mol% (left) and the lowest arginine content 0.3 mol% (right).

The most essential step for the chemical reduction process presented here (Figure 2) is the degassing of the heated sugar solution, which helps improving the reduction power of the monosaccharide.[^41^, ^42^, ^43^] The copper source and arginine solutions are added and mixed with further degassing and heating. When this solution is also heated up, the reaction can be started by adding sodium hydroxide increasing the pH value up to 14 for a short time, leading to a stepwise reduction of the copper(II) ions. This is indicated by a visible colour change (Figure 3) from light blue to dark blue, yellowish brown and dark brown. This corresponds to the formation of copper hydroxide followed by the tetrahydroxy copper(II) complex. Next the copper is reduced first to copper(I) oxide, resulting eventually in pure copper and a brown solution from the Maillard products. During the reaction the pH value decreases eventually to 9, due to the reaction to hydroxy compounds and consumption during the Amadori rearrangement. The brown solution indicates a successful Maillard reaction, in which the monosaccharides react with the arginine.^[44]^ The influence of the pH value is shown in **Fig S2**. During this reaction copper is reduced by two separate single electron transfers.[^42^, ^43^] Since this reaction is directly influenced by the concentration of the arginine, it was varied in the range from 0.3 mol% to 27 mol% which is approximately four times the saturation concentration at room temperature. Therefore, overview measurements with SEM (Figure 4) were performed, showing the reaction products after using the highest and lowest arginine content as an example (full collection in **Figure S3**).

**Figure 5:**
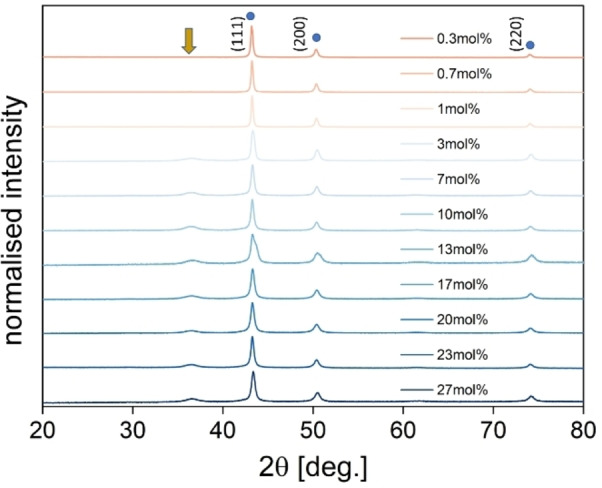
PXRD pattern of the resulting copper particles obtained with different arginine concentrations. Blue dots indicate the reflections of the copper phase, and the yellow arrow indicates the most significant reflection of the copper(I) oxide phase.

From the image on the left in Figure 4 it is clearly visible that the size of the particles is much smaller when the content of arginine is high. The lower the added content of arginine to the reaction is, the bigger are the resulting particles. The more crystal nuclei are present in a solution which is dependent on the arginine concentration, the smaller are the resulting particles. This trend is observable over the whole concentration range depicted in Figure S3. To proof the phase purity of the resulting particles PXRD measurements were performed for all samples obtained with different concentrations of arginine (Figure 5).

Beside the reflections resulting from the copper phase (JCPDS No. 00–4‐836) at 43.1°, 50.4° and 74.0° another reflection is visible at around 36.5° for the experiments with higher arginine content. It is visible for all experiments starting from 3 mol% arginine or higher. This reflection is related to a copper(I) oxide phase (JCPDS No. 00–5‐667) in these samples. However, since this reflection has low intensity, it might result from a spontaneous oxidation of the sample since they are exposed to air before each PXRD measurement.[^45^, ^46^] The samples prepared with less arginine do not show the copper(I) oxide phase. As can be observed from the SEM images, the resulting particles are bigger having less surface area, which leads to a higher stability against oxidation by oxygen in the air. To proof this point a model experiment was conducted in which the particles were synthesised with strict exclusion of oxygen from air. Here, the results (**Figure S4‐S5**) show a pure metallic copper phase after the reaction for galactose. Ribose on the other hand seems not to be able to perform the reduction even under inert conditions.

**Figure 6:**
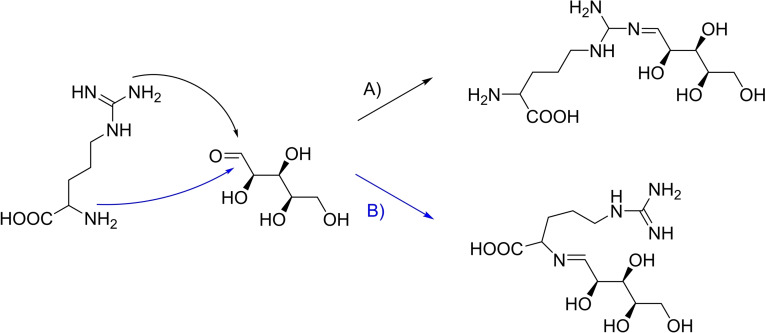
Two possible ways of the attack of arginine on the carbonyl group of xylose in the Amadori rearrangement.

Arginine has an advantage compared to other amino acids because it is able to perform the Amadori rearrangement followed by a Maillard reaction in two ways[^45^, ^46^, ^47^, ^48^] schematically depicted in Figure 6.

Because of the two containing amino functionalities in the amino acid moiety and in the side chain the attack on the carbonyl compound can be performed in principle both ways.[^49^, ^50^, ^51^] This has two possible advantages. First the rate of the attack of the amino compound on the carbonyl group is highly dependent on the basicity of the amino group. Therefore, an improvement of the reaction can be achieved by using either one or the other amino group depending on the conditions during the reaction. Moreover, similar behaviour is possible for the complexation of the copper ions. They can form a complex with the guanidinium moiety but also with the amino acid part of the molecule.[^52^, ^53^] It is also possible to form a complex with a larger ring between both functional groups. In either way, the copper ions are captured and can possibly form the crystal nuclei for the nanoparticle synthesis.

Since it was proven that it is possible to prepare nanoparticles with the adaptation of the previously reported environmentally friendly method, the influence of the used monosaccharide was investigated, too. It is known that copper ions form different complexes with carbohydrate depending on the kind of carbohydrate.^[54]^

**Figure 7:**
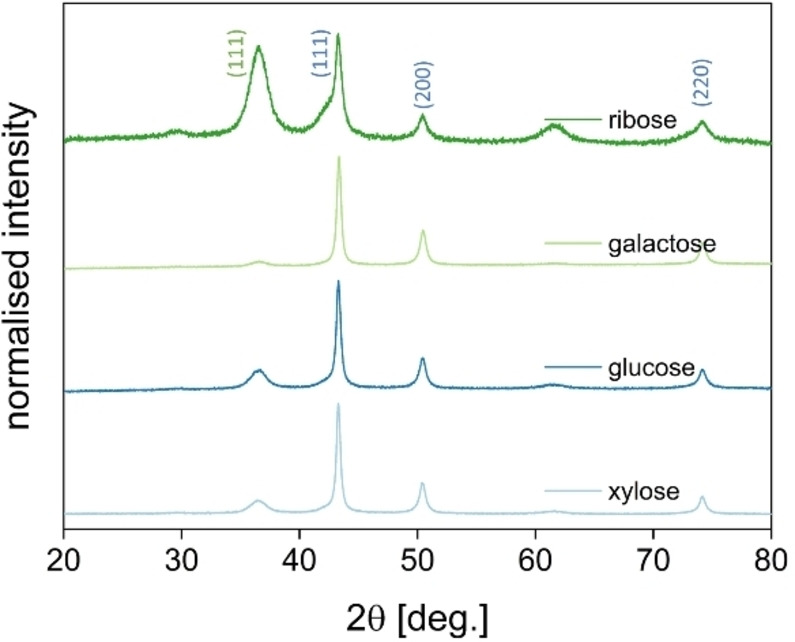
PXRD pattern of the resulting copper particles with different monosaccharides as reducing agent. The blue indexation is corresponding to metallic copper, green for copper(I) oxide.

Therefore, in addition to the aldopentose xylose, also ribose was tested, as well as the aldohexoses galactose and glucose. All four compounds were added in the same molar amount during the synthesis, with an arginine content of 14 mol% with respect to the monosaccharide. First, again PXRD measurements (Figure 7) were performed.

**Figure 8:**
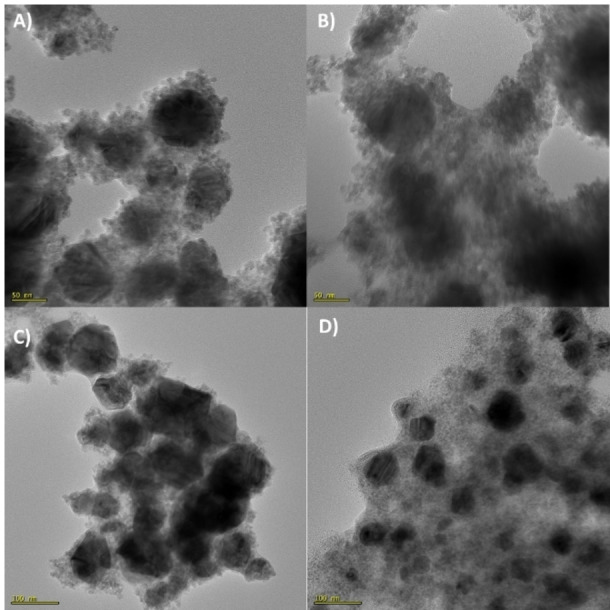
TEM images of the resulting copper particles with A) xylose (120k x magnification), B) glucose (120k x magnification), C) galactose (80k x magnification) and D) ribose (80k x magnification) as reducing agent.

From the PXRDs it is clearly visible that in terms of phase purity the reaction with galactose gives the best results, even better than the reaction with xylose, which had been chosen in the previous study. These findings are supported by EDS measurements in **Fig S6**. Nonetheless, also with glucose copper is obtained, the low amount of copper(I) oxide could again be related to the contact with air while measuring PXRD. Ribose does not seem to be suitable for this application. Even though the SEM images (**Figure S7**) look promising, high intensities of the copper(I) oxide phase can be observed. For all measurements a broadening of the copper(I) oxide reflections can be observed. This again indicates an oxidation of the copper in contact with air, which is more pronounced for the smallest particles in the sample due to a higher reactive surface. To analyse the morphology of the nanoparticles TEM images (Figure 8) were recorded.

The images show similar results for all copper nanoparticles prepared by the different reducing agents. All samples show few spherical nanoparticles with a larger size just below 100 nm. Besides, all samples show a high amount of smaller also spherical nanoparticles in the size between 3–16 nm (average diameter and standard deviation in **Table S1**) with a narrow size distribution (**Figure S8)**. Due to the high phase purity the sugar galactose was preferred, also showing good results in the TEM images. Further tests were conducted to get an idea about the stability of the nanoparticles. Therefore, PXRD was measured again after 12 weeks. From the pattern (**Fig S9**) it can be seen that no visible degradation occurs and still a nearly pure metallic copper phase can be measured.

From this it can be concluded that the combination of galactose as reducing agent and arginine as amino compound in this synthesis result in phase pure metallic copper nanoparticles. The optimal composition and conditions of the synthesis are summarised in **Table S1**.

## Conclusions

This work presents a new way of preparing copper nanoparticles in an environmentally friendly way. The particles were synthesized in an aqueous solution with only naturally occurring reagents performing the reduction of a copper(II) salt. Inspired by nature, the Amadori rearrangement and a following Maillard reaction was utilised by bringing a naturally occurring amino acid and a sugar to reaction. The resulting nanoparticles were investigated by PXRD, SEM and TEM measurements showing a pure copper phase under inert conditions and nanoparticles with sizes down to 3–16 nm in diameter. The combination of galactose and arginine was demonstrated to result in the most favourable products regarding the phase purity and size of the particles. However, also xylose and glucose as reducing agents showed good results. All three sugars are naturally abundant and easily accessible.

## Experimental Section

*Materials*: All chemicals were used without further purification. D‐(+)‐xylose (>99 %), D‐(−)‐ribose (>98 %), D‐(+)‐glucose (>99.5 %) were purchased from Sigma Aldrich / Merck. D‐(+)‐galactose (>98 %) and L‐arginine (>98.5 %) were purchased from Carl Roth, copper(II) chloride (99 %) from fisher scientific and sodium hydroxide (>97 %) from VWR.

*Copper reduction*: In a round bottom flask 60 ml deionised water is heated to 100 °C. Continuously argon is bubbled through the reaction mixture during the whole reaction time. After the solvent is heated to the required temperature 12 ml of 2.8 M monosaccharide solution (xylose, ribose, galactose and glucose) are added and kept under stirring at 100 °C for 15 minutes. Afterwards, 12 ml of 1.3 M copper(II) chloride solution and 4 ml arginine solution in various concentrations in the range from 0.3 mol% to 27 mol% are added and mixed for 5 more minutes. To start the reaction 12 ml of 10 M sodium hydroxide solution are added. The reaction is successful when a visible colour change from blue to light blue, followed by light brown to dark brown, occurs. After maximum 30 minutes reaction time the resulting product is washed by centrifugation followed by washing with water and ethanol three times. All parameters are summed up in **Table S2**.

*Characterisation*: SEM images are taken with a Hitachi SU8020 using 2 kV acceleration voltage. X‐ray diffractograms were recorded using Panalytical Aeris with CuK1 radiation. The high‐resolution transmission electron microscopy (HR‐TEM) images are taken with a JEOL Jem F‐200 C‐transmission electron microscope equipped with a Gatan OneView‐in‐situ 4 K camera at an acceleration voltage of 200 kV. The samples were drop‐casted on TED Pella ultrathin carbon film on lacey carbon support 300 mesh gold grids.

## Supporting Information

Supporting Information is available from the Wiley Online Library or from the author.

## Conflict of Interests

The authors declare no conflict of interest.

1

## Supporting information

As a service to our authors and readers, this journal provides supporting information supplied by the authors. Such materials are peer reviewed and may be re‐organized for online delivery, but are not copy‐edited or typeset. Technical support issues arising from supporting information (other than missing files) should be addressed to the authors.

Supporting Information

## Data Availability

The data that support the findings of this study are available from the corresponding author upon reasonable request.
