# Supplementary material for: Green Synthesis of Copper Nanoparticles utilising the Maillard Reaction
Source: Chemistry. 2025 Feb 12;31(18):e202404314. doi: 10.1002/chem.202404314 (PMC11937877; doi:10.1002/chem.202404314)
Supplement: Supplementary file 1 — Supporting Information [file CHEM-31-e202404314-s001.pdf]

Chemistry–A European Journal

Supporting Information

Green Synthesis of Copper Nanoparticles utilising the Maillard Reaction

Lukas Mielewczyk, Virginia Liebscher, Julia Grothe,* and Stefan Kaskel

Supplementary Information

Green Synthesis of Copper Nanoparticles utilising the Maillard Reaction

Lukas Mielewczyk, Virginia Liebscher, Julia Grothe, Stefan Kaskel

[a] *Department of Inorganic Chemistry, Technische Universität Dresden. Bergstrasse 66, 01069 Dresden.*

Modified synthesis of copper nanoparticles under fully inert conditions

In a sealed beaker with reflux condenser 60 ml deionised water are heated to 100 °C. Continuously argon is bubbled through the reaction mixture during the whole reaction time. After the solvent is heated to the required temperature 12 ml of 2.8 M galactose solution is added and kept under stirring at 100 °C for 15 minutes. Afterwards, 12 ml of 1.3 M copper(II) chloride solution and 4 ml arginine solution in various concentrations like mentioned above in the range from 0.3 mol% to 27 mol% are added and mixed for 5 more minutes. To start the reaction 12 ml of 10 M sodium hydroxide solution are added. The reaction is accompanied by a visible colour change from blue to light blue, followed by light brown to dark brown occurs. After maximum 30 minutes reaction time the resulting product is transferred with syringes into an argon tent for the washing steps. Water and ethanol used for washing are degassed for at least 30 minutes before the use. When transferring the samples to the centrifuge they were sealed with parafilm and just opened under the tent again. The sample was dried under vacuum and measured under exclusion of air in scotch tape.

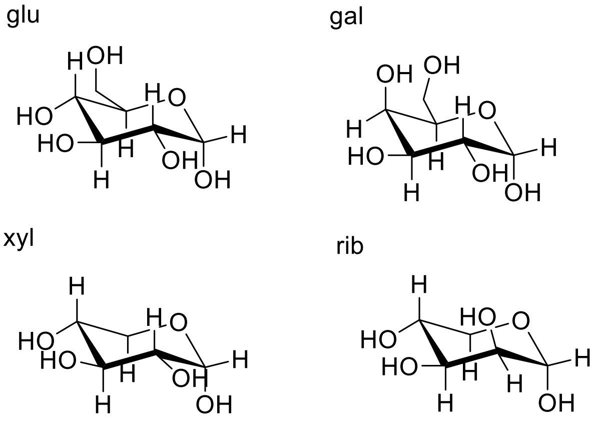

Figure S1. *Structures of the four used monosaccharides. Each of them in alpha pyranose form.*

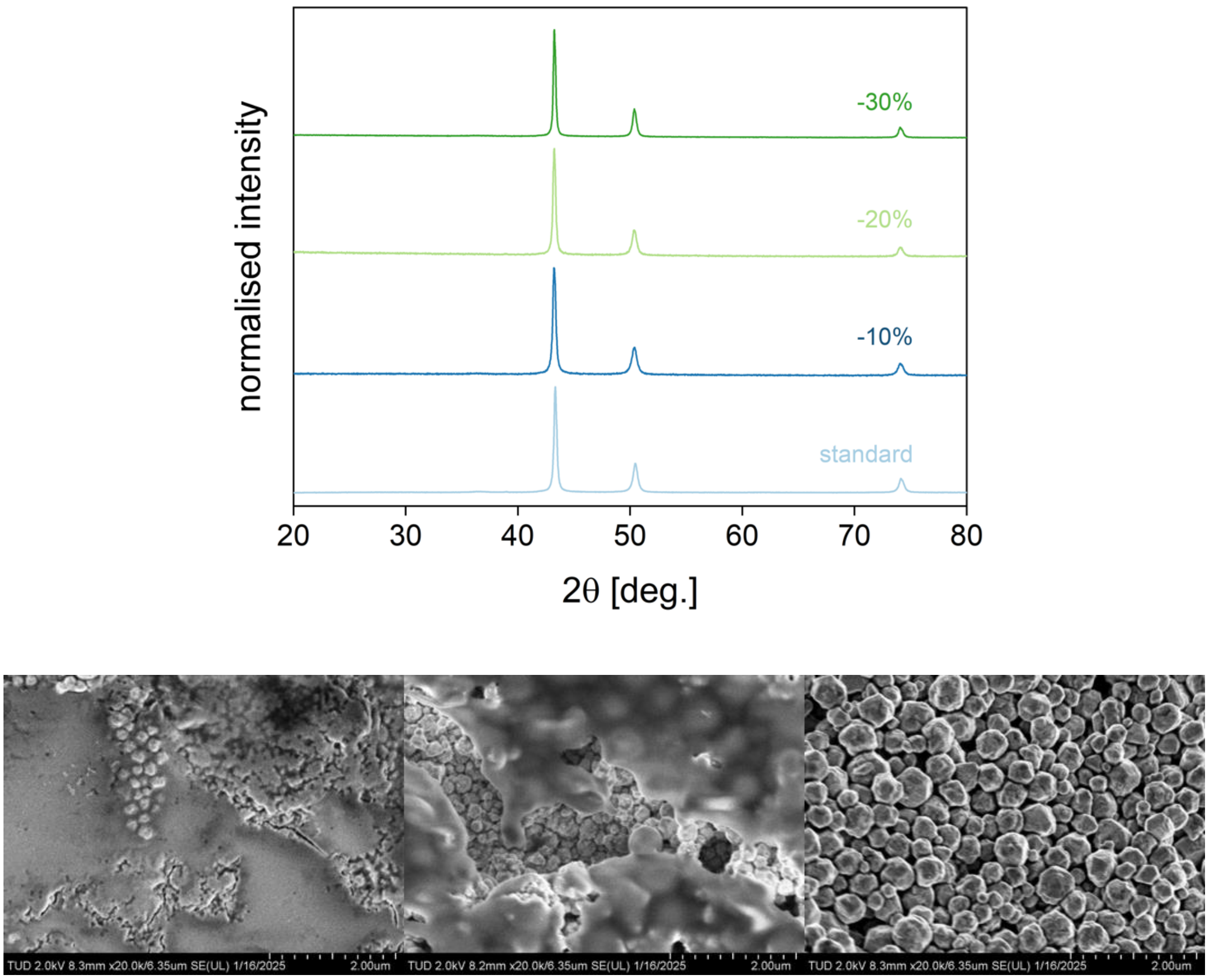

Figure S2. *Top: PXRD pattern of a synthesis conducted with different sodium hydroxide concentrations to show the influence of the pH value. Bottom: SEM images of the samples with less sodium hydroxide with (left) -10%, (middle) -20% and (right) -30%.*

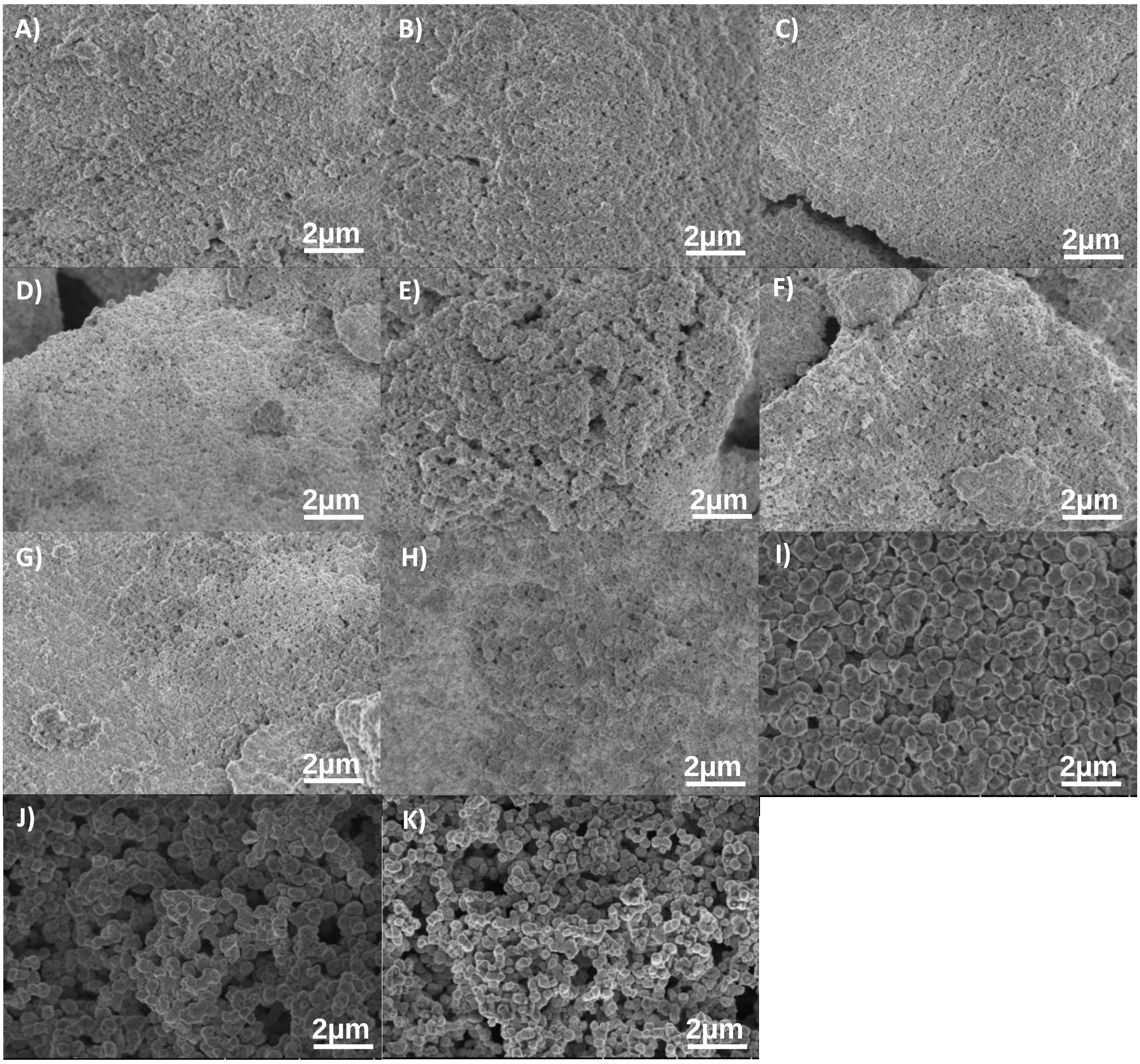

Figure S 3: *SEM images of the copper powder resulting from the reaction with A)27 mol%, B) 23 mol%, C) 20 mol%, D) 17 mol%, E) 13 mol%, F) 10 mol%, G) 7 mol%, H) 3 mol%, I) 1 mol%, J) 0.7 mol% and K) 0.3 mol% arginine.*

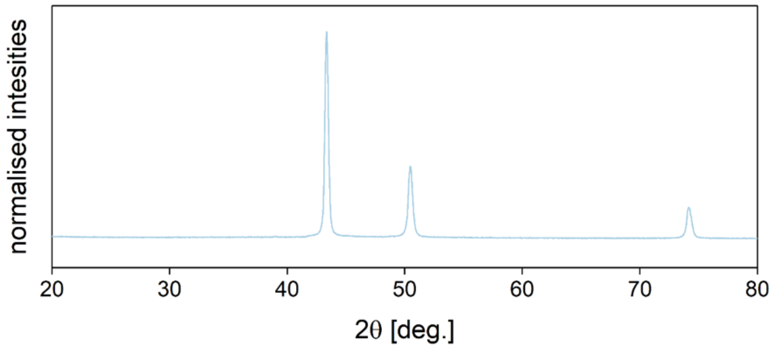

Figure S4*. PXRD pattern of copper nanoparticles prepared under inert conditions and strict exclusion of oxygen using galactose as reducing agent. The product contains only a pure metallic copper phase and no side products.*

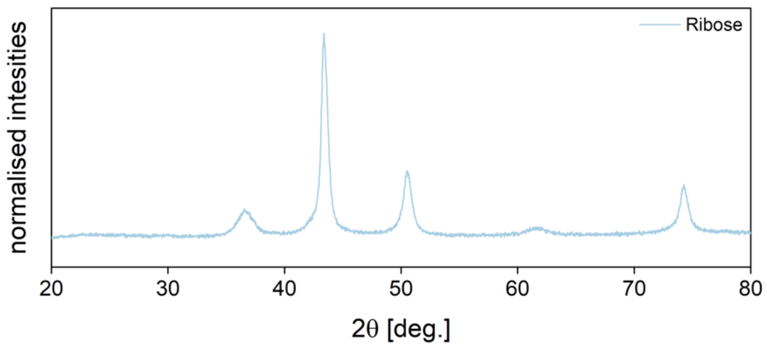

Figure S5. *PXRD pattern of copper nanoparticles prepared under inert conditions and strict exclusion of oxygen using ribose as reducing agent. Copper(I) oxide is observable with a peak at around 36.5°.*

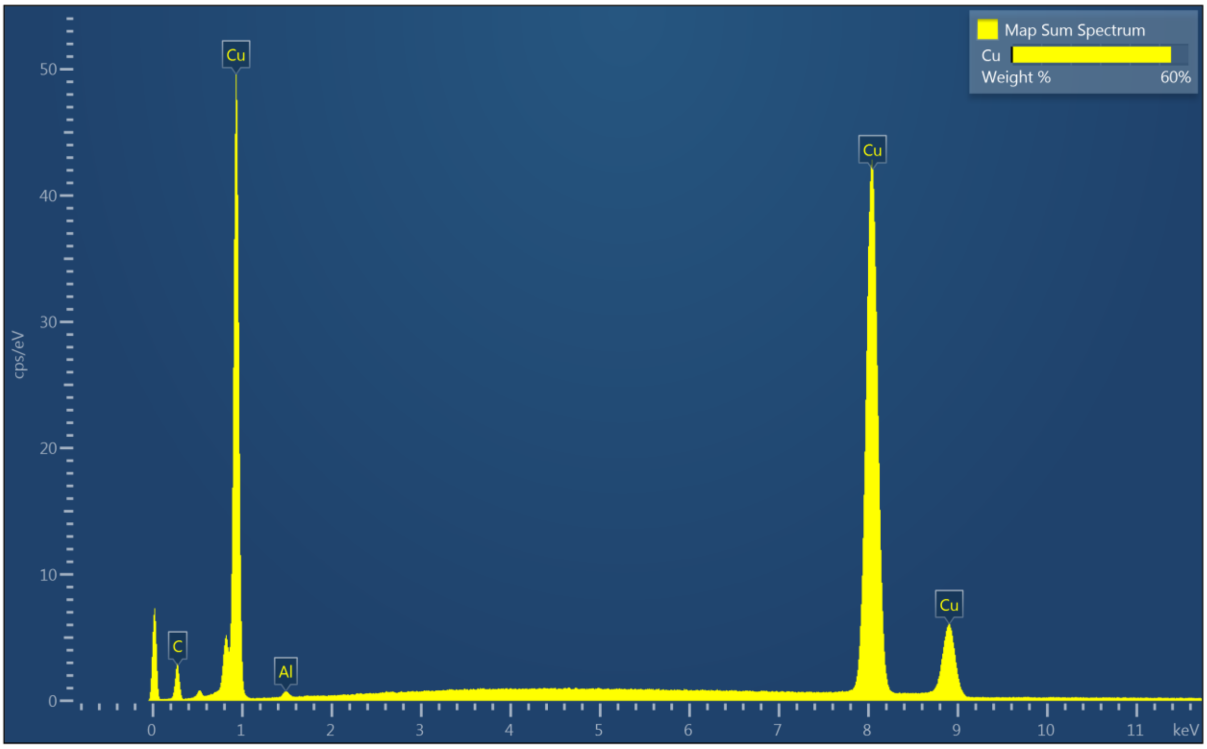

Figure S6. *EDS measurement of copper nanoparticles prepared with galactose as reducing agent. No oxygen was observed. Carbon and Aluminium are measured due to the carbon pad and the aluminium carrier used during the measurement.*

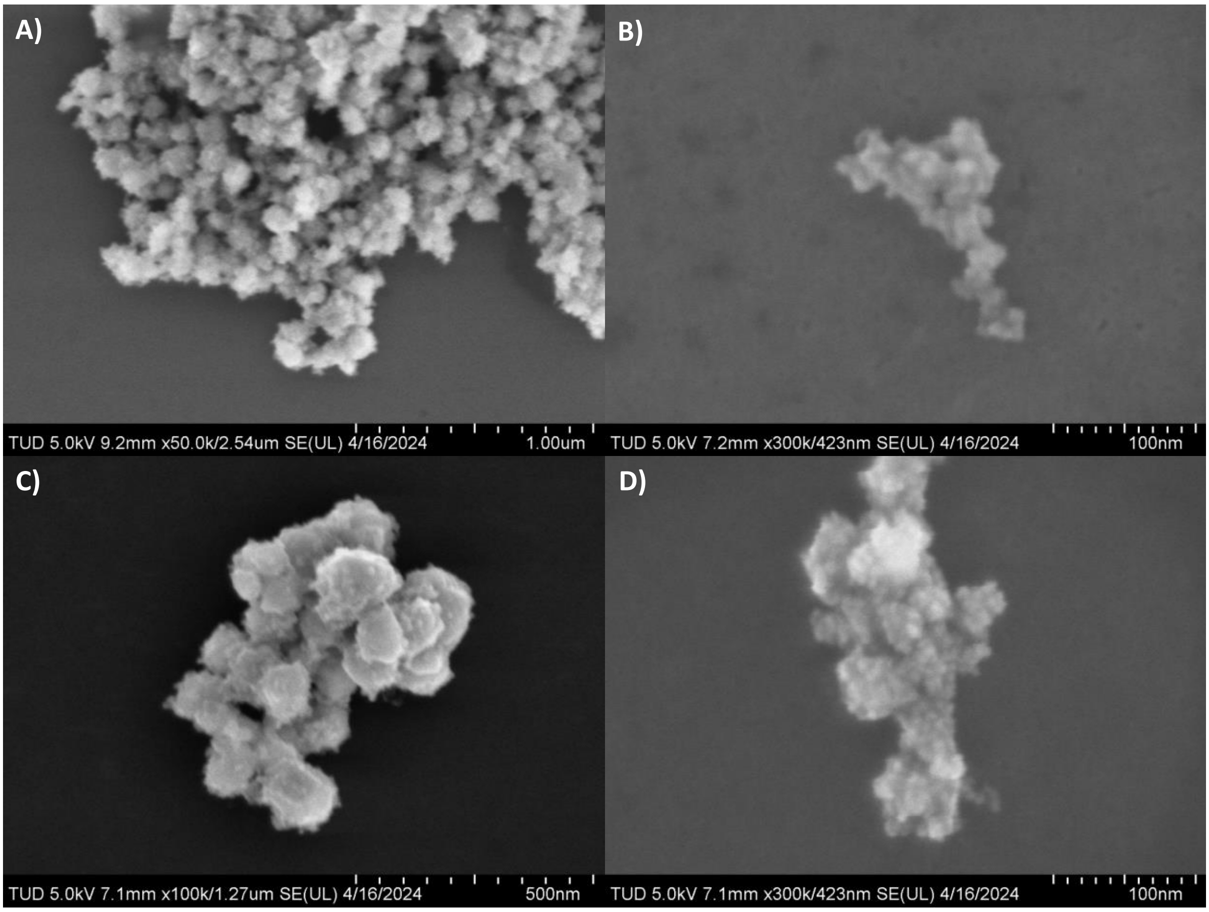

Figure S 7*: SEM images of the resulting copper particles with A) xylose, B) glucose, C) galactose and D) ribose as reducing agent.*

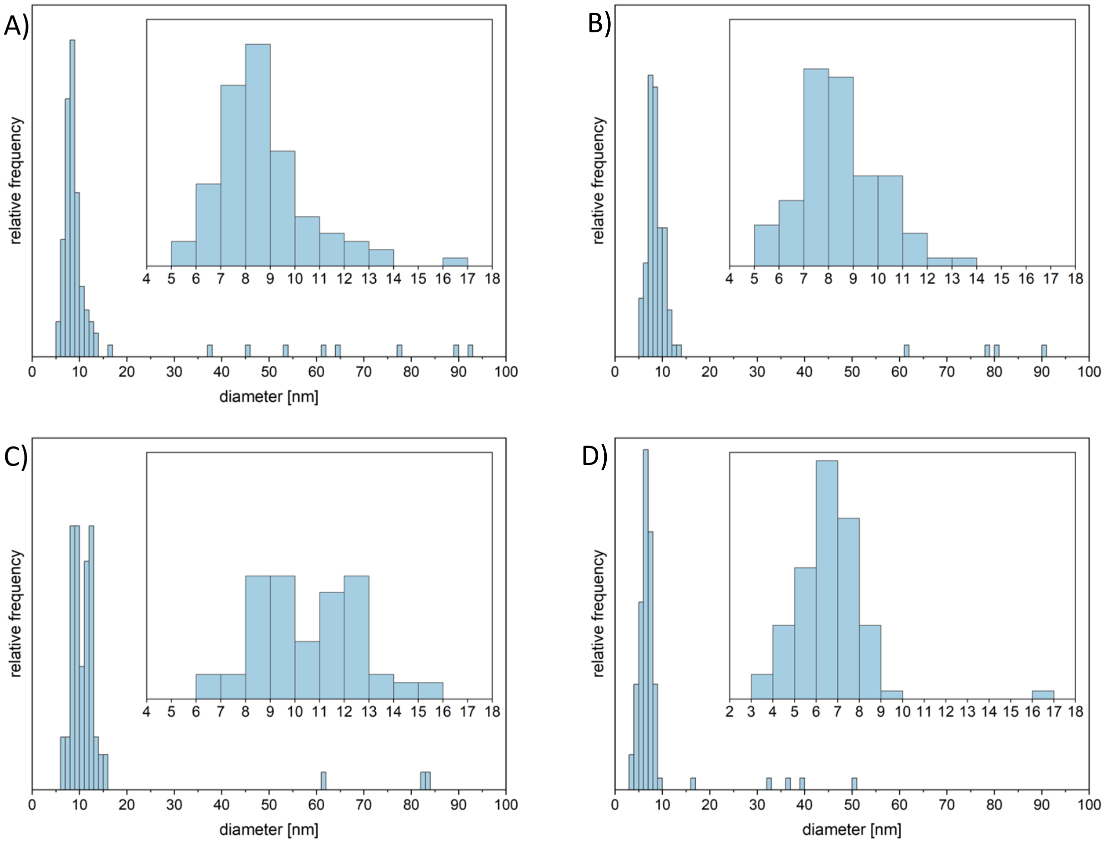

***Figure S8**: Histograms regarding the distribution of the diameters of the copper nanoparticles made with A) xylose, B) glucose, C) galactose and D) ribose as reducing agent. For each diagramm 200 particles were taken into account.*

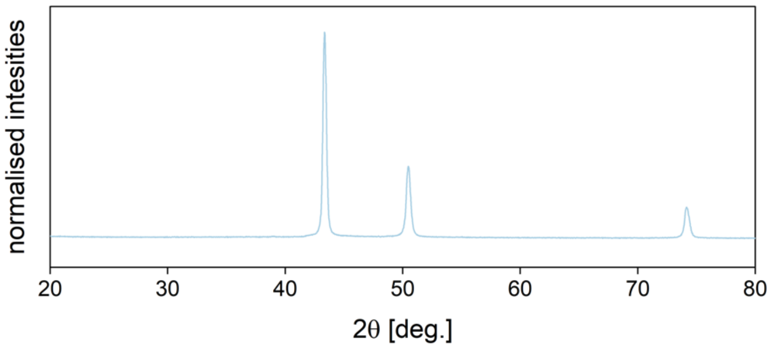

***Figure S9.** PXRD pattern of a sample after 12 weeks of preparation to give insight into the phase stability while storing.*

Table S1. Average diameter and standard deviation of copper nanoparticles regarding the reducing agent used.

Reducing agent	Average diameter and standard deviation
galactose	12.97 ± 12.69 nm
xylose	13.21 ± 16.37 nm
glucose	11.49 ± 14.49 nm
ribose	8.01 ± 7.05 nm

Table S2. Parameters for the synthesis of copper nanoparticles. All solutions listed are prepared in deionised water.

Constituents	Volume / concentration
Monosaccharide	12 ml / 2.8 M
Copper(II)chloride	12 ml / 2.8 M
Arginine	4 ml / 8.4 M
Sodium hydroxide	12 ml / 10 M
**Operation conditions**	
Temperature	100°C
Atmosphere	argon
Reaction time	30 minutes
